# Local Hyperemia, or Excess of Blood in Dental Tissues

**Published:** 1868-05

**Authors:** B. T. Spellman


### DENTAL REGISTER.

VOL. XXII.] MAY, 1868. [No. 5.

### Original Communications.

### LOCAL HYPEREMIA, OR EXCESS OF BLOOD IN DENTAL TISSUES.

Read before the Northern Ohio Dental Association, May 5th, 1868.

BY B. T. SPELLMAN.

THAT Dental pulps are not only subject to this pathological condition, but *often* become so, every Dental practitioner has learned to his sorrow. To prove this fact would be a work of supererogation, and unworthy of this occasion. The proximate causes and pathological changes ultimating in inflammation, as well as the effect of the same on the pulp, is the task I have, in my humble way, chosen to perform.

It is not sufficient to say that the proximate cause is the exposure of the pulp. First, because the advancement of my hearers in pathological science will not tolerate so superficial a view; and second, because it is not always true. We often have congestion, inflammation, suppuration and death, without exposure. The same too often follows unfortunate Dental operations; as when a filling impinges on the pulp, as well as when a conductor of heat and cold is in contact with this tissue.

God in his wisdom has so organized the animal economy, that when any violence, or the least encroachment is made on any tissue, the blood, the great purveyor of plasma, flows in increased volume to the point of attack, to reinforce the garrison and repel the invader. This is determination of blood. It is sent here to conserve the life and well-being of the part to which it flows; exalting at the same time its sensibility.

The physical characteristics of this form of hyperemia are increased warmth, redness and size of tissue. The vital, are secretion, sensibility and nutrition. This is an abnormal condition, which if continued, ultimates in congestion and inflammation; and if it be local, as in a Dental periosteum, and uncontrolled, suppuration and abscess. And if in the pulp, *death*.

Determination is not always a characteristic of disease. Hypertrophy is always the result of increased nutrition, as is increased muscle, previously diminished by disease or inactivity. The latter condition is the result of an increased flow of blood to a part—"with motion increased;" but the former is the result of an "excess of blood with a like increase of motion." To convey this increased quantity of blood, the arteries, capillaries and veins are enlarged. This is usually the result of increased motion, and physical force of the heart. As for example, when the blood is forced to the head, lungs and neck. And also the congestion of internal organs, as manifested in some stages of fever. But local determination often takes place without any increased motion of the heart, or any other, except a negative force. And as this is the force (if I may so call it) which operates in determination of blood to Dental periosteum and pulp, I must give it more than a passing notice.

The force which I have here called negative, pathologists denominate atony. Tonicity is a property possessed by many of the tissues of the body; as in voluntary and involuntary muscle. It is by virtue of this fôrce that the air tubes expel their contents, and the urinary bladder performs the same office by contracting on its contents. It is a tonic, or contractile force. The force which so often defeats the operation for strabismus, by drawing the eyeball past the axis of vision, when the opposing muscle is severed; the force which holds limbs out of place when dislocated; the force which "resides in the middle coat of the arteries and causes them to expel their contents after death, even to complete exhaustion or collapse."

Any force or agency which will diminish this tonicity, will cause the arteries to dilate, and the arteries in this vicinity will force their contents with motion to the enlarged vessels; and the increased flow of blood will increase the caliber of the capillaries connected with the dilated organ, as well as the veins which receive the blood from them. Thus we have an "excess of blood in a part;" and thus we have "determination of blood in a part" without any increased action of the heart.

Now it will be seen at once, that any agent which will diminish the tonicity of the arteries in the Dental pulp, will increase the flow of blood to the same. And if obstruction takes place in the canal, we have congestion, inflammation and death. I should observe here that recent writers on pathology hold that the nerves, particularly the sympathetic, have a great influence in causing hyperemia.

Having thus briefly spoken of local determination, with increased motion of the blood, I have arrived at a point where I can connect this pathological condition with local hyperemia, with diminished motion of blood; and show the connection of both diseased conditions in the production of local congestion

Hyperemia, with diminished motion of blood in a part, is congestion.

I have already shown the influence of tonicity, or atony in the production of determination of blood to a part. It will now be seen that a minus quantity of the same force is one of the agents which conduce to congestion. The capillaries have much less tonicity than the arteries, and the veins still less than the former.

Obstruction and congestion always occur in the capillaries and veins, and this obstruction is the cause of diminished motion of the blood; and one of the distinguishing characteristics of congestion. Unlike determination, the arteries are not increased in size, but the capillaries are. When determination of blood to a part resolves itself into congestion, the arteries, in consequence of their greater tonicity, contract; while the veins and capillaries enlarge for the want of this force. And if the determination continues for an undue length of time, the arteries and veins become enervated by over excitement, and suffer a loss of tone, and in consequence become distended. Over excitement of these vessels may, therefore, be recognized as a cause of congestion. Cold, by contracting the vessels of the surface, will cause the blood to retire from the same, and cause internal congestion. And this receding blood will flow to the part in which the vessels have the least tonicity. If this condition is not relieved, fatal inflammation of internal organs will follow; as congestive fevers, consumption, etc. But as I am treating of local congestion, these remarks are out of place. Now it is plain that the arrest and accumulation of blood in a part is congestion. And this condition of the vessels favors the accumulation of the pale corpuscles. They will cohere one to another, and to the walls of the vessels, obstructing the onward flow of blood; and hence congestion.

All tissues are nourished by the blood through the media of the capillaries, or absorption. And each tissue of the animal organism has the power of taking, or assimilating its own peculiar nourishment from the blood. Now capillaries in which there is diminished motion or congestion, and more so where we find complete obstruction, are enervated and lose their tonicity for want of nourishment. The tissues also which depend momentarily on the blood, through the media of the congested vessels, are impoverished and enfeebled. The veins will share the same fate as the capillaries; and the waste or effete matter of the parts, which it is their office to eliminate or take up by endosmosis will remain to poison, and further weaken the impoverished part; reducing it to the same condition that the entire system would be in if we were compelled to re-inhale, at each successive inspiration, the carbonic acid which we have just exhaled.

To remove the obstruction and invigorate the part, nature sends in an increased flow of the nutrient fluid, and we have the characteristic phenomenon of inflammation ; namely, excess of blood in a part with "motion, partly increased and partly diminished." And if the vigor of the system is unable to overcome this inflammation, the physician must resort to his revellents; which if unsuccessful, will compel us to combat the ultimate consequences of congestion, inflammation, suppuration and abscess.

The Dental pulp is almost wholly a plexus of blood vessels, and the connective tissue is traversed by a very great number of capillaries ; which if exposed by decay, will lose much of their tonicity, from the enervating influence of the secretions of the mouth alone, even if these are in their normal condition. But where we have decaying dentine, we will have gases and acids evolved, or set free to act on the pulp. The decomposition of animal and vegetable deposits in the cavities of decay, as well as in many other parts of the mouth, are fruitful sources of irritating, and I might say, poisonous agents. Nor is this all the tortures which this little silent monitor has to suffer. There are ever active thermal changes present to complete and hasten on the hateful pathological condition, viz.: congestion.

This condition is sometimes, yes, very often, complicated with another of congestion, not spoken of in the books. It is a mechanical or strangulated congestion. It occurs when part of the pulp is forced out into the cavity of decay. This being the result of congestion, it can never precede it.

As in other tissues, we can have in the pulp determination of blood without congestion, and *vice versa.* But a continuance of either shortly ultimates in inflammation.

The foramen at the apex of a tooth is so small compared to the pulp, that very slight causes acting on the vessels will cause a loss of tonicity sufficient to produce congestion of the capillaries and veins. And as congestion often ruptures these vessels in other parts of the body, it is clear that the same results may follow in the pulp. Hence we have effusion, strangulation (already spoken of), inflammation, suppuration and death. It is my opinion, that no more blood passes into an exposed pulp than into one in its normal condition; but the motion of the blood is retarded, or wholly arrested in the veins and capillaries. In consequence, we get effusion in two ways; first, by the rupture of the vessels already alluded to; and second, by a pressure on the vessels, forcing the liquor sanguineous through the walls of the vessels, while the fibrin and the red corpuscles remain. But before the last phenomenon takes place, the liquor sanguineous passes through or past the aggregated corpuscles, until complete obstruction follows the heaping together of the more solid portions of the blood.

At, or before the occurrence of the last mentioned phenomenon, the third form of hyperemia asserts its sway; and inflammation is the godhead of the trinity. I say trinity, because this form of hyperemia embraces the other two, already alluded to, viz.: determination, and congestion. It is the reserved force of the animal economy; a force behind the stagnant blood to move it onward in its proper channels.

Inflammation has the same physical characteristics as the other forms of hyperemia, differing only in degree. But being one remove further from a physiological condition, they are much more marked. We have more heat, more swelling, more redness, and more pain. The latter is the result in the Dental pulp, as well as in other tissues, of the pressure on the nerve or nerves, caused by the swelling of surrounding parts. If we have determination of blood to the Dental pulp, we have enlarged arteries, and corresponding capillaries and veins. The result is pain. If we have congestion, then distended capillaries and veins, and consequently painful pressure. When inflammation takes place, we have not only increased pressure, but more exalted sensibility and heat. Odontalgia is not always, therefore, a disease of the Dental nerves, but derangement of the circulation. And it is by acting on these vessels, that tooth-ache is cured by astringents. They constringe or lessen the size of the vessels, and thus relieve the nerve from the pressure.

This subject is one on which volumes have been written. And if I should extend this paper to ten times its present length, which I might, the subject would not be exhausted. And feeling in a charitable, rather than in an aggressive mood, I will extend to the Association the charity of my silence, and a final period.

